# Regulatory Clearance and Approval of Therapeutic Protocols of Transcranial Magnetic Stimulation for Psychiatric Disorders

**DOI:** 10.3390/brainsci13071029

**Published:** 2023-07-04

**Authors:** Gonçalo Cotovio, Fabiana Ventura, Daniel Rodrigues da Silva, Patrícia Pereira, Albino J. Oliveira-Maia

**Affiliations:** 1 Champalimaud Research and Clinical Centre, Champalimaud Foundation, 1400-038 Lisbon, Portugal; goncalo.cotovio@neuro.fchampalimaud.org (G.C.); fabi.ventura4@gmail.com (F.V.); daniel.silva@research.fchampalimaud.org (D.R.d.S.); patricia.fernandes.pereira@fundacaochampalimaud.pt (P.P.); 2 NOVA Medical School, Faculdade de Ciências Médicas, NMS, FCM, Universidade NOVA de Lisboa, 1169-056 Lisbon, Portugal; 3 Departamento de Psiquiatria e Saúde Mental, Centro Hospitalar de Lisboa Ocidental, 1449-005 Lisbon, Portugal; 4 Departamento de Psiquiatria e Saúde Mental, Centro Hospitalar e Universitário de Coimbra, 3000-075 Coimbra, Portugal; 5 Portuguese Red Cross Health School, 1300-125 Lisbon, Portugal

**Keywords:** transcranial magnetic stimulation, regulatory agencies, clearance, approval, psychiatric disorders

## Abstract

Non-invasive brain stimulation techniques (NIBS) have been widely used in both clinical and research contexts in neuropsychiatry. They are safe and well-tolerated, making NIBS an interesting option for application in different settings. Transcranial magnetic stimulation (TMS) is one of these strategies. It uses electromagnetic pulses for focal modulate ion of neuronal activity in brain cortical regions. When pulses are applied repeatedly (repetitive transcranial magnetic stimulation—rTMS), they are thought to induce long-lasting neuroplastic effects, proposed to be a therapeutic mechanism for rTMS, with efficacy and safety initially demonstrated for treatment-resistant depression (TRD). Since then, many rTMS treatment protocols emerged for other difficult to treat psychiatric conditions. Moreover, multiple clinical studies, including large multi-center trials and several meta-analyses, have confirmed its clinical efficacy in different neuropsychiatric disorders, resulting in evidence-based guidelines and recommendations. Currently, rTMS is cleared by multiple regulatory agencies for the treatment of TRD, depression with comorbid anxiety disorders, obsessive compulsive disorder, and substance use disorders, such as smoking cessation. Importantly, current research supports the potential future use of rTMS for other psychiatric syndromes, including the negative symptoms of schizophrenia and post-traumatic stress disorder. More precise knowledge of formal indications for rTMS therapeutic use in psychiatry is critical to enhance clinical decision making in this area.

## 1. What Is Transcranial Magnetic Stimulation and Repetitive Transcranial Magnetic Stimulation?

Non-invasive brain stimulation (NIBS) corresponds to a group of techniques that are used to modulate brain activity and do not require invasive procedures such as surgery [1]. Neuronal activity can be modulated through changes in the electrical and chemical activity of neurons, with the aim of enhancing or suppressing specific regions or circuits in the brain, potentially changing the associated functions and behaviors [2]. Transcranial magnetic stimulation (TMS) is a NIBS strategy that uses electromagnetic pulses to modulate the neuronal activity of targeted regions [3]. In TMS, a magnetic field with an intensity of around 1.5 Tesla is produced by an electrical current flow of up to 8000 A through a coil placed on the scalp, resulting in the depolarization of neurons in the underlying cortex [4,5]. Different types of coils determine the distinct shapes of the magnetic field—the figure-of-eight coil, the most commonly used, allows for penetration of up to a depth of around 2.5 cm [6]. Coils with winding patterns, double-cone configurations, or other shapes allow for deeper penetration, potentially reaching subcortical structures [7], in what is called deep transcranial magnetic stimulation (dTMS). Other stimulation parameters, such as pulse shape, current direction, stimulation target, and stimulation intensity and frequency, can also be modeled to alter the response to TMS [8]. Stimulation intensity is typically individualized, based on the resting motor threshold (rMT), i.e., the lower intensity to elicit a motor response when applied over the motor cortex [9,10].

Stimulation frequency is related to long-term changes in brain activity induced by TMS. Repetitive transcranial magnetic stimulation (rTMS) is used in clinical settings to induce longer lasting changes in brain activity by repeatedly applying electromagnetic pulses for a certain period using a predetermined frequency. High-frequency (typically 10 Hz or higher) or low-frequency (typically 1 Hz) rTMS protocols will most frequently result in an increase or decrease in cortical excitability, thought to result from mechanisms of long-term potentiation (LTP) and long-term depression (LTD), respectively [8]. Indeed, cortical excitability is a phenomena arising from the interaction of multiple neurotransmission mechanisms [11], with excitation supported by glutamate, and inhibition through gamma-aminobutyric acid, among other neurotransmitter systems [12,13]. LTP and LTD are lasting neuroplastic phenomena impacting neural excitability and have been consistently implied to be present in the putative mechanisms of action of TMS (please see Suppa et al. [14] for further details). More recently, more complex rTMS protocols have been developed, such as intermittent theta burst stimulation (iTBS), based on the theta patterns of physiological brain functioning. iTBS consists of the application of bursts of three pulses at high frequency (50 Hz), with an inter-burst interval of 200 ms. This is thought to produce faster but also more long-lasting changes, when compared to standard high-frequency protocols [8].

## 2. How Is Transcranial Magnetic Stimulation Used?

Since its first introduction in 1985 by Anthony Barker [15], TMS has been widely used both in research and for clinical purposes [16]. In the former, TMS has been used, for example, to study potential neurophysiologic mechanisms associated with psychiatric or neurologic conditions and is a valuable tool for improving current pathophysiologic models for such conditions, possibly with the aim of defining innovative therapeutic strategies [17,18,19,20]. In the clinical context, it is mainly used as a treatment option for certain neuropsychiatric disorders. The neurobiological mechanisms underlying the therapeutic effects of TMS for psychiatric disorders is still a matter of debate, with several hypotheses proposed, including neuroendocrine, neurochemical, and neuroprotective changes in specific brain circuits (please see Post and Keck [21] for further details). 

Currently, there are several stimulator devices and coils, with specific stimulation protocols designed and tested for the treatment of well-defined neuropsychiatric disorders [22]. Response rates vary depending on several factors, in addition to TMS protocol, such as the treated condition (e.g., major depressive episode, obsessive compulsive disorder, post-traumatic stress disorder) and co-adjuvant treatment strategies [23]. Indeed, TMS clinical response has been reported to be as high as 78% when using a specific accelerated protocol for treatment-resistant depression [24], illustrating the need to invest in understanding the factors associated with enhancing and sustaining the clinical effects of TMS. Such factors may be protocol-related, including optimal duration or stimulation frequency, or patient-related [25,26,27], such as clinical profile [25,26,27], personality traits [28], brain plasticity [29], cortical excitability [10,30], specific circuitry disfunction [31,32], or hemispheric activity imbalance [30]. These findings may favor individualized treatment protocols [24,33,34] or the combination of therapeutic strategies (e.g., TMS and psychotherapy) [35,36] to increase treatment efficacy. 

Importantly, rTMS is a safe and well-tolerated NIBS strategy that does not require anesthesia or a prolonged recovery period after each session. Adverse events (AE) are mostly mild and self-limited, the most common being scalp pain or discomfort (40%) or headaches (40%) at the beginning of stimulation [23]. Other less common but also mild AE have been reported, including gastrointestinal discomfort (5–22%), muscle twitching (0–20.6%), dizziness (0–16.7%), insomnia (4.5–7.6%), difficulty concentrating (0–41.7%), hypomania (5%), tinnitus (0–11%), skin pain (1–8.5%), or fainting (1%) [23,37]. The potentially most severe AE are TMS-induced seizures, typically also self-limited, and extremely rare (0.01–0.1%), with an incidence comparable to that of most psychotropic medications [23,38,39]. Available data do not support additional risks of major AE in specific patient populations, such as those suffering from psychiatric disorders/conditions, at least when considering standard TMS protocols [38]. Indeed, expert opinion and safety guidelines support the use of approved and/or recommended stimulation parameters (e.g., stimulation intensity or frequency) to decrease the likelihood of these events [38].

## 3. Transcranial Magnetic Stimulation Therapeutic Protocols for Psychiatric Disorders

### 3.1. TMS Protocols for Major Depressive Disorder (with/without Comorbid Anxiety)

While risk management and quality assurance are guaranteed by manufacturers, external regulatory agencies audit, certify, and approve or clear the use of TMS devices and/or protocols in clinical practice and research. In Europe, this is the responsibility of the Conformitè Europëenne (CE) Mark (CE Mark per EU directive 93/42/EEC), while in the United States of America, this role belongs to Food and Drug Administration (FDA). In fact, both agencies have reviewed and approved or cleared TMS devices and protocols for use as treatment strategies in different neuropsychiatric disorders. Additionally, evidence-based recommendations by clinical experts have also been published to support the clinical use of rTMS, based on the most up-to-date clinical evidence [40].

The first study on the therapeutic use of rTMS in psychiatry was published in 1995, involving patients with medication-resistant depression [41]. Meanwhile, many other studies, including large multi-center trials, demonstrated the efficacy and safety of rTMS in treatment-resistant depression (TRD). In 2008, the FDA cleared the first rTMS protocol to treat depression, which consisted of high-frequency (10 Hz) rTMS delivered over the left-dorsolateral prefrontal cortex (L-DLPFC) for just under 38 min per session, with a stimulation intensity of 120% of the rMT, across 30 sessions (5 days a week for 6 weeks) [42]. In the study leading to this decision, 301 medication-free patients diagnosed with TRD were randomized to sham or active TMS groups of the L-DLPFC [42], with response rates according to the Montgomery–Asberg depression rating scale significantly superior in the active vs. sham group (24% vs. 12%) [42]. Importantly, active TMS was well tolerated and adverse events were generally mild and limited to transient scalp discomfort (11%) or pain (36%) [42]. Regarding the treatment target, the DLPFC has been associated with several cognitive functions and has been shown to play a major role in cognitive control among patients with depression [43]. Indeed, the DLPFC, particularly on the left, is functionally impaired in major depression which, in addition to its superficial position, makes it an optimal target for rTMS [44]. More recently, novel forms for delivering rTMS to the left DLPFC have emerged, including dTMS [45]. Here, a helmet-like coil, designated as the H1-coil, stimulates the lateral prefrontal regions bilaterally, more intensely in the left DLPFC, according to electric field models [45]. Different studies have confirmed its efficacy, with response rates reported to be between 38 and 55% [46,47,48]. The FDA has cleared the use of dTMS with the H1-coil to treat depression (see Table 1 for further details).

In 2018, the FDA cleared a further iTBS protocol to treat depression, based on a non-inferiority trial comparing iTBS with the standard 10 Hz rTMS protocol for TRD [49]. In this study, 414 patients were randomly allocated to receive either of these two active TMS protocols [49]. Not only were response rates similar between iTBS and 10 Hz rTMS (49% vs. 47%) but their AE profiles also overlapped, with headaches being the most common side-effects (65% vs. 64%) [49]. iTBS is delivered in 2 s bursts consisting of three pulses at a frequency of 50 Hz, with an inter-burst interval of 200 ms (i.e., 5 Hz), resulting in much briefer sessions, lasting only 3 min and 9 s. Similarly to the standard rTMS protocol, stimulation intensity is 120% of rMT, the target is the L-DLPFC, and treatments are performed on weekdays for up to 30 daily sessions (please see Table 1 for a detailed description of the FDA-cleared TMS protocols to treat major depressive episodes). Importantly, several TMS devices have also received CE certification covering use in treatment-resistant depression, but the associated treatment protocols are less clearly defined and typically available from TMS device manufactures. Since 2018, at the Champalimaud Foundation Neuropsychiatry Unit (CF-NPU), we have treated patients with TMS for major depressive episodes, using approved devices and protocols, with the majority of treatments performed using the iTBS protocol. In accordance with available data, at the end of treatment, approximately half and one-third of our patients were classified as responders and remitters, respectively.

The efficacy of rTMS for major depressive episodes has been consistently confirmed in several meta-analyses [50,51,52], with response and remission rates varying from 39.5 to 70% and 16.6 to 76.9%, respectively [53]. Importantly, the antidepressant response resulting from TMS was also shown to be long-lasting, with 53% and 46% of those who respond to an acute rTMS treatment cycle having sustained responses lasting 6 and 12 months after treatment, respectively [25]. Protocols, other than those mentioned above, such as low-frequency rTMS targeting the right DLPFC (R-DLPFC), as well as continuous TBS or bilateral stimulation targeting both L- and R-DLPFC, have also been shown to be clinically effective but have lower recommendation levels and are yet to be approved by regulatory agencies [40]. Further details on alternative rTMS protocols for depression, including low-frequency rTMS or cTBS to the right DLPFC, or bilateral stimulation, are available in different systematic reviews and metanalysis [50,51,52] as well as expert guidelines and recommendations [40], frequently used to decide on off-label use (please see Table 2 for details). At our clinical unit, and according to most recent expert recommendations [40], we have offered off-label low-frequency R-DLPFC rTMS to patients diagnosed with depression that did not tolerate L-DLPFC protocols and/or had important comorbidities, such as severe anxiety or obsessive compulsive disorder (OCD). In fact, in 2021, and more recently in 2022, the FDA cleared two different rTMS devices and protocols to treat depression with comorbid anxiety symptoms, both targeting the L-DLPFC with high frequency stimulation. This decision was based on cumulative evidence from different trials that have shown a significant decrease in these symptoms when treating major depressive episodes [42,46,47,54]. Indeed, these studies reported a reduction in anxiety symptoms with effect sizes varying between 0.4 and 2.5, favoring TMS treatment [42,46,47,54]. No major safety concerns were highlighted [42,46,47,54]. TMS devices have also received CE certification that covers their use in depression with comorbid anxiety disorders. This decision was supported by the evidence of equivalent efficacy of two different protocols: bilateral sequential rTMS of the L-DLPFC and R-DLPFC or unilateral low frequency rTMS of the R-DLPFC [55]. Moreover, due to its safety profile regarding side effects and drug interaction, TMS is positioned as a treatment option for special populations diagnosed with a depressive episode, including older adults, peripartum depression, and bipolar disorder. In older adults diagnosed with depression, TMS has been consistently shown to be effective and tolerable [27,56]. In peripartum depression, TMS appears to be effective and safe, but there is still no recommendation or approved protocol [57]. Finally, there is evidence to support the use of rTMS in bipolar depression, although there are mixed results with different protocols and more robust studies are needed [58]. Studies identifying which rTMS protocols are more effective for which conditions, as well as to identify the potential predictors of treatment response, are also needed.

### 3.2. TMS Protocols for Obsessive Compulsive Disorder

While, clinically, TMS was initially used mainly in the context of depression, recently it has also been used as a treatment for obsessive compulsive disorder (OCD). Dysfunction in cortico–striate–thalamo–cortical (CSTC) circuits has been hypothesized to be associated with the development of symptoms of OCD [60]. This association has been consistently supported across research modalities [61,62,63,64], such as neuroimaging or cognitive paradigms, including response inhibition, set shifting, or decision making, which are impaired in individuals diagnosed with OCD [64,65]. Not surprisingly, rTMS protocols for OCD treatment have aimed to modulate CSTC circuits. According to a recent network meta-analysis, which included 22 randomized controlled trials (RCTs), the most effective rTMS strategies for treatment of OCD are low frequency rTMS applied over the DLPFC (mean difference = 6.3) or the supplementary motor area (mean difference = 4.2), high frequency rTMS applied over the DLPFC (mean difference = 3.8), and high frequency dTMS applied over the anterior cingulate cortex and dorsomedial prefrontal cortex (ACC/mPFC; mean difference = 4.3) [66]. Importantly, all rTMS treatment protocols were similar to sham in what tolerability is concerned [66]. 

Recently, a multi-center RCT in 99 patients diagnosed with OCD demonstrated efficacy of high frequency dTMS over the ACC/mPFC relative to sham stimulation [67]. Response rates were 38% and 11% for active dTMS and sham, respectively [67]. Indeed, the results of this study lead to a decision by the FDA to clear TMS as co-adjuvant treatment for OCD in adult patients (please see Table 1 for a detailed description of the FDA-cleared TMS protocol to treat OCD). Importantly, this protocol was developed to include systematic symptom provocation at the beginning of each session of brain stimulation. This approach consists of eliciting a moderate level of distress before each TMS session by prompting individualized symptoms, a process thought to allow for the reconsolidation of fear and distressing memories into long-term memories, which may thus be disrupted by brain stimulation [35,68]. Similarly, TMS devices have received CE certification covering their use as co-adjuvant treatment for OCD, using the same protocol. Here, as mentioned above for depression, treatment protocols for TMS devices with CE certification are less clearly defined. At the CF-NPU, we recently initiated an rTMS program for OCD treatment, according to the above-mentioned FDA-approved protocol.

### 3.3. TMS Protocols for Other Psychiatric Disorders/Conditions

The use of TMS in the treatment of other neuropsychiatric disorders has been supported by clinical evidence, expert guidelines (Table 2), and in some cases, regulatory agencies (please see Table 1 for further details on the TMS protocols cleared by the FDA). Among substance use disorders, high frequency dTMS, targeting the lateral prefrontal cortex and insula bilaterally, has demonstrated a reduction in cigarette consumption with an abstinence rate of 44% at the end of treatment, leading to FDA clearance for smoking cessation [69]. In the context of misuse of other psychoactive substances, high frequency rTMS over the L-DLPFC induced long-lasting reductions in cocaine use [70,71], with TMS devices receiving a CE certification that covers its utilization for substance use disorders [70,71].

Regarding post-traumatic stress disorder (PTSD), several studies have suggested that different TMS protocols may reduce symptoms, but regulatory approval is lacking. Most protocols used for PTSD apply unilateral high frequency rTMS to either the left or right DLPFC, with one protocol using dTMS to the mPFC [72]. In one RCT, 58 patients diagnosed with PTSD were randomized to receive 20 Hz-rTMS either over the R-DLPFC, the DLPFC bilaterally, or sham stimulation [73]. Active rTMS consisted of 2400 pulses/session over the R-DLPFC or 1200 pulses over the R-DLPFC followed by 1200 pulses over the L-DLPFC [73]. While there was no significant difference between R-DLPFC and bilateral DLPFC, both of the active TMS protocols were more efficient than the sham (41.2 and 62.5 vs. 0% of responders, respectively). Although a specific protocol is yet to be approved, studies have been more consistent in suggesting that high frequency TMS over the R-DLPFC yields better results than other approaches [74], resulting in a formal recommendation for this treatment in expert guidelines (Table 2), with potentially better results if concomitant psychotherapy is considered [36]. In fact, as mentioned above, this combined approach has been supported not only in PTSD [75] but also in other conditions, such as OCD [67], as it may help modulate the reconsolidation of long-term memories as well as cognitive processes [76].

Studies analyzing the potential role of TMS for the positive symptoms of schizophrenia are still scarce. A 2020 systematic review found that most studies comprise small sample sizes and focus only on auditory hallucinations, making it difficult to draw any robust conclusion about TMS treatment in this context [77]. Low frequency rTMS over the left temporoparietal cortex has been proposed to have a promising role for auditory verbal hallucinations [77,78], with other rTMS protocols targeting different cortical regions also tested, with mixed findings [77,78]. Overall, studies focusing on TMS treatment for positive symptoms of schizophrenia, particularly auditory hallucinations, were limited and heterogenous [40,77,78]. Hence, available evidence is not sufficiently consistent to recommend the widespread use of TMS for the positive symptoms of schizophrenia [40]. Nevertheless, further details on the use of rTMS for these indications are available in systematic reviews [77,78] and expert guidelines and recommendations [40], frequently used to decide on off-label use. On the other hand, TMS has also been explored as a treatment option for negative and cognitive symptoms of schizophrenia. Those are usually persistent and very difficult to treat, being one of the most important predictors of poor global functioning and poor prognosis [79]. Recent studies have identified that structural and functional changes in prefrontal cortical areas, as well as their (dys)connection with striatal regions, are associated with negative symptoms of schizophrenia [80], making the PFC a potential target for TMS. In fact, a recent network meta-analysis involving 48 RCTs and 2211 patients suggested that high frequency rTMS protocols over the L-DLPFC may improve the negative symptoms of schizophrenia [81]. Hence, although they are currently not approved, such rTMS protocols may be promising in the treatment of the negative symptoms of schizophrenia.

While TMS has also been explored in other psychiatric disorders, such as attention deficit hyperactivity disorder or autism spectrum disorder, current evidence is poor and/or negative, leading to a lack of approval by regulatory authorities and an absence of formal recommendations in expert guidelines [40]. Finally, TMS has also been studied to treat other not strictly psychiatric disorders, such as the cognitive symptoms of Alzheimer’s disease and tinnitus, but are yet to be approved by regulatory authorities [40]. Further details on the use of TMS for these or other non-psychiatric indication are available in systematic reviews [82,83] and expert guidelines and recommendations [40], frequently used to decide on off-label use. A summary of recommendations based on the level of evidence of TMS for the treatment of psychiatric disorders, as presented in Lefaucheur et al., is presented in Table 2.

## 4. Conclusions

The use of transcranial magnetic stimulation has been increasing, given that it has been consistently shown to be an effective, safe, and well tolerated treatment alternative for several psychiatric disorders [22]. In fact, in the conditions for which it is approved, TMS is not only effective in reducing symptoms, but it also improves the quality of life and functional outcomes, including social functioning, occupational performance, and overall well-being [84,85,86]. Moreover, economic evidence from several countries has suggested that TMS is also a cost-effective strategy for TRD when compared to pharmacotherapy, and possibly also in comparison to electroconvulsive therapy [87,88]. Hence, it is not surprising that the use of TMS is growing worldwide [89]. It is thus critical that both the providers and prescribers of this treatment have precise knowledge of approved treatment indications and protocols, to allow for adequately informed decision making by patients and their physicians [38]. Here, we summarized information regarding the TMS treatment protocols that are cleared and/or approved by international regulatory agencies for treatment-resistant depression, obsessive compulsive disorder, and certain substance use disorders. Moreover, we discussed the promising data to support use of TMS in other psychiatric conditions, including PTSD or negative symptoms of schizophrenia, albeit still in the absence of regulatory approval and with a need for better powered and multi-center clinical trials. Finally, we underlined wide and emerging research opportunities in the field of TMS for psychiatric disorders, with a potential for clinically meaningful impacts [90], including the search for novel indications [91] and stimulation targets [34], the optimization of treatment protocols [24], and the investigation of neuroplastic mechanisms underlying treatment response [29].

Providing appropriate information to patients is an ethical obligation of medical providers to allow for informed decision making and consent, which we propose should be obtained in writing in the case of rTMS. In this process, clinicians must describe relevant risks and benefits, not only for indications approved/cleared by regulatory authorities but also for off-label TMS protocols [38]. The full disclosure of the treatment plan in the informed consent process is critical, particularly if off-label TMS protocols, acceptable depending on country-specific regulations and the quality of evidence, are considered [38]. Further details on the ethical issues of TMS therapeutic use are available in Rossi et al. [38], while expert recommendations that can be consulted to decide on off-label use are available in Lefaucheur et al. [40]. These and other guidelines provide practical recommendations for clinicians and researchers planning to implement therapeutic TMS in psychiatric settings, including treatment plan, response monitorization, and the integration of TMS with other treatment modalities [38,40]. This manuscript is an additional instrument to aid this process through the provision of information on protocols with regulatory approval by North American and European authorities.

## Figures and Tables

**Table 1:** Description of FDA-cleared transcranial magnetic stimulation protocols to treat psychiatric disorders.

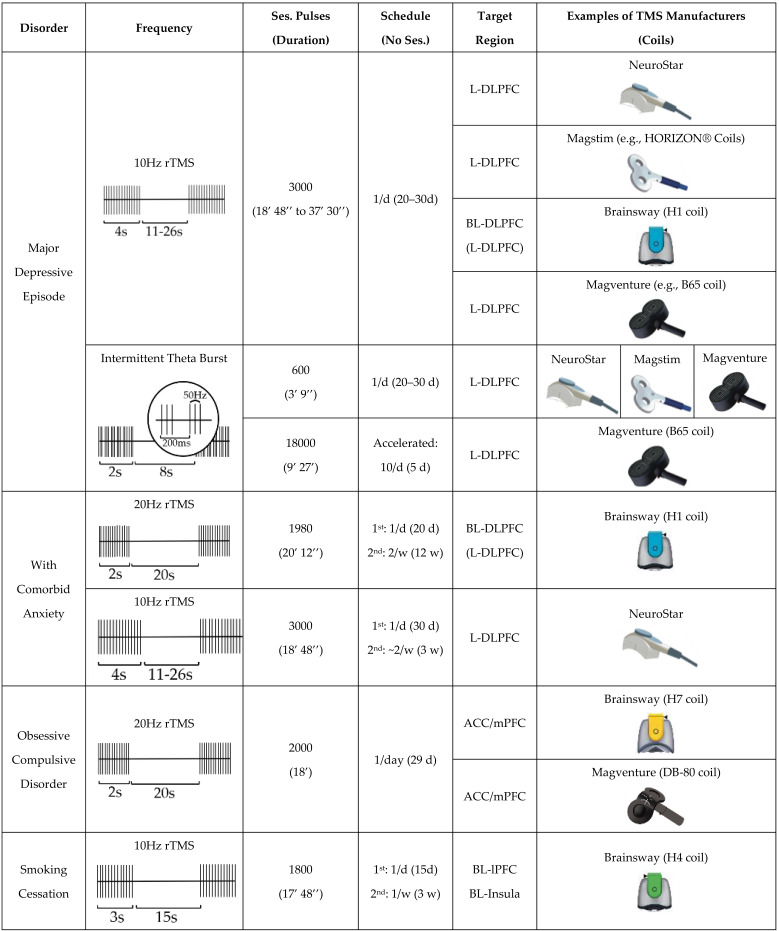

’—minutes; ’’—seconds; ACC—anterior cingulate cortex; BL—bilateral; CE—Conformitè Europëenne; d—day; DLPFC—dorsolateral prefrontal cortex; FDA—Food and Drug Administration; Hz—Hertz; L—left; lPFC—lateral prefrontal cortex; mPFC—medial prefrontal cortex; No—number; rTMS—repetitive transcranial magnetic stimulation; s—seconds; Ses.— sessions; TMS—transcranial magnetic stimulation; w—weeks. This table reflects the protocols that have been cleared by FDA for TMS use in the treatment of psychiatric disorders. Treatment protocols for TMS devices that have received CE certification are less clearly defined. FDA clearance and CE certification are further described in the text, with the supporting documents of CE certification decisions obtained from TMS device manufactures whenever necessary.

**Table 2:** Summary of recommendations of TMS in psychiatric disorders.

Major Depressive Episode	Definite antidepressant effect of HF-TMS of the left DLPFC (Level A)
	Probable antidepressant effect of LF-TMS of the right DLPFC (Level B) and probably no differential antidepressant effect between right LF-TMS and left HF-TMS (Level B)
	Definite antidepressant effect of rTMS of the DLPFC in unipolar depression (Level A), but no recommendation for bipolar depression
	Antidepressant effect of rTMS of the DLPFC is probably additive to the efficacy of antidepressant drugs (Level B) and possibly potentiating (Level C)
PTSD	Possible effect of HF-TMS of the right DLPFC (Level C)
Auditory hallucinations	Possible effect of LF-TMS of the left TPC (Level C)
Negative symptoms of schizophrenia	Probable effect of HF-TMS of the left DLPFC (Level B)
Addiction and craving	Possible effect of HF-rTMS of the left DLPFC on cigarette craving and consumption (Level C)

HF—high-frequency; LF—low-frequency; TMS—transcranial magnetic stimulation; DLPFC—dorsolateral prefrontal cortex; TPC—temporoparietal cortex; PTSD—post-traumatic stress disorder. Level A (‘‘definitely effective or ineffective”) required at least two Class I studies or one Class I study and at least two Class II studies; Level B (‘‘probably effective or ineffective”) required at least two Class II studies or the combination of one Class I or II study and at least two Class III studies; and Level C (‘‘possibly effective or ineffective”) required at least two Class III studies or any combination of two studies of different Classes I, II, or III. Table adapted from Lefaucheur, J.P. et al. Evidence-based guidelines on the therapeutic use of repetitive transcranial magnetic stimulation (rTMS). *Clin. Neurophysiol.* **2014**, *125*, 2150–2206 [59].

## Data Availability

Not applicable.
