# Correction: Cotovio et al. Regulatory Clearance and Approval of Therapeutic Protocols of Transcranial Magnetic Stimulation for Psychiatric Disorders. *Brain Sci.* 2023, *13*, 1029

**DOI:** 10.3390/brainsci14020153

**Published:** 2024-02-02

**Authors:** Gonçalo Cotovio, Fabiana Ventura, Daniel Rodrigues da Silva, Patrícia Pereira, Albino J. Oliveira-Maia

**Affiliations:** 1 Champalimaud Research and Clinical Centre, Champalimaud Foundation, 1400-038 Lisbon, Portugal; goncalo.cotovio@neuro.fchampalimaud.org (G.C.); fabi.ventura4@gmail.com (F.V.); daniel.silva@research.fchampalimaud.org (D.R.d.S.); patricia.fernandes.pereira@fundacaochampalimaud.pt (P.P.); 2 NOVA Medical School, Faculdade de Ciências Médicas, NMS, FCM, Universidade NOVA de Lisboa, 1169-056 Lisbon, Portugal; 3 Departamento de Psiquiatria e Saúde Mental, Centro Hospitalar de Lisboa Ocidental, 1449-005 Lisbon, Portugal; 4 Departamento de Psiquiatria e Saúde Mental, Centro Hospitalar e Universitário de Coimbra, 3000-075 Coimbra, Portugal; 5 Portuguese Red Cross Health School, 1300-125 Lisbon, Portugal

**Missing Citation**

In the original publication [1], **Lefaucheur, J.P.; André-Obadia, N.; Antal, A.; Ayache, S.S.; Baeken, C.; Benninger, D.H.; Cantello, R.M.; Cincotta, M.; de Carvalho, M.; De Ridder, D.; et al. Evidence-based guidelines on the therapeutic use of repetitive transcranial magnetic stimulation (rTMS). *Clin. Neurophysiol*. 2014, 125, 2150–2206**, was not cited. The citation has now been inserted in Table 2 legend and should read:

“Table adapted from Lefaucheur, J.P. et al. Evidence-based guidelines on the therapeutic use of repetitive transcranial magnetic stimulation (rTMS). *Clin. Neurophysiol.* **2014**, *125*, 2150–2206 [59]”.

With these corrections, the order of some references has been adjusted accordingly.

The correct references are listed below:

59. Lefaucheur, J.P.; André-Obadia, N.; Antal, A.; Ayache, S.S.; Baeken, C.; Benninger, D.H.; Cantello, R.M.; Cincotta, M.; de Carvalho, M.; De Ridder, D.; et al. Evidence-based guidelines on the therapeutic use of repetitive transcranial magnetic stimulation (rTMS). *Clin. Neurophysiol*. **2014**, *125*, 2150–2206.
